# Prevalence of microalbuminuria and its associated cardiometabolic risk factors in Korean youth: Data from the Korea National Health and Nutrition Examination Survey

**DOI:** 10.1371/journal.pone.0178716

**Published:** 2017-06-02

**Authors:** Heeyeon Cho, Jae Hyun Kim

**Affiliations:** 1 Department of Pediatrics, Samsung Medical Center, Sungkyunkwan University School of Medicine, Seoul, Republic of Korea; 2 Department of Pediatrics, Seoul National University Bundang Hospital, Seongnam, Republic of Korea; Universita degli Studi di Perugia, ITALY

## Abstract

**Background:**

Microalbuminuria is a known early predictive factor for renal and cardiovascular diseases, not only for patients with diabetes mellitus or hypertension but also in the general population. However, the prevalence and risk factors associated with microalbuminuria in Korean youth are unknown.

**Objectives:**

The aims of this study are to evaluate the prevalence of microalbuminuria and the association between microalbuminuria and obesity or cardiometabolic risk factors in Korean children and adolescents without diabetes.

**Methods:**

This study examines data obtained from the Korea National Health and Nutrition Examination Survey (between 2011 and 2014). It includes a total of 1,976 participants aged between 10 and 19 years (boys 1,128 and girls 848). Microalbuminuria was defined as a urine albumin-to-creatinine ratio (UACR) of ≥ 30 mg/g and < 300 mg/g. Association between microalbuminuria and the risk factors for cardiometabolic diseases including insulin resistance was evaluated.

**Results:**

The prevalence of microalbuminuria was found to be 3.0% in Korean children and adolescents over this time period. The mean UACR for non-obese youth was significantly greater than that found in obese youth (3.2 ± 0.1 mg/g in the non-obese group vs. 2.1 ± 0.2 mg/g in the obese group; *P* < 0.001). In multiple logistic regression analysis, microalbuminuria was associated with hyperglycemia (OR 2.62, 95% CI 1.09–6.30) and hemoglobin A1c (OR 3.34, 95% CI 1.09–10.17) in the non-obese group and hypertension (OR 14.10, 95% CI 1.12–177.98) and HbA1c (OR 6.68, 95% CI 1.87–23.95) in the obese group.

**Conclusions:**

The prevalence of microalbuminuria is not prominent in obese children and adolescents. Our findings demonstrated that the presence of hypertension and hyperglycemia was associated with microalbuminuria. Especially Hemoglobin A1c was associated with microalbuminuria in youths regardless of weight status. Microalbuminuria in pediatric population can be a helpful marker for the risk of cardiovascular disease.

## Introduction

Microalbuminuria occurs when urine albumin is significantly greater than normal, and is diagnosed when urinary albumin excretion is between 30 and 300 mg/day, or when the microalbumin/creatinine ratio is between 30–300 μg/mg in random urine [1]. It has been shown to be an early predictive factor for renal and cardiovascular diseases, not only in patients with diabetes mellitus or hypertension but also in the general population [2]. Additionally, increased urinary albumin is thought to be a consequence of renal disease [3]. The mechanisms that make microalbuminuria a predictor of future cardiovascular events remain poorly understood; it is thought that the main cause is related to endothelial dysfunction [4]. This potential causal connection is explained as resulting from the increased systemic albumin permeability caused by endothelial dysfunction, and that hemodynamic abnormalities interact with additional factors, such as lipids abnormalities, systemic inflammation, increased activity of renin-angiotensin-aldosterone system, and prothrombin factors, which together can lead to widespread organ damage [4]. It is possible that endothelial dysfunction is innate, because increased albumin excretion has been observed in neonates and toddlers with a high variability of individual [3]. There remains no consensus, however, as to whether inborn endothelial dysfunction can cause renal and/or cardiovascular disease, or if an acquired risk factor, such as obesity, plays a major role in the development of microalbuminuria and organ damage.

Several studies in obese children and adolescents have established specific risk factors that are associated with microalbuminuria. These include body mass index (BMI), waist circumference (WC), triglyceride (TG), sex and metabolic syndrome in children and adolescents [5–8]. The association between obesity and microalbuminuria in children from these prior studies, however, remains unclear, and a thorough analysis of the general pediatric population is lacking. Additionally, previous studies that have supported a positive association between microalbuminuria and cardiovascular risk factors, such as hypertension in children, sometimes arrived at contradictory conclusions, and it remains questionable as to whether microalbuminuria is an independent predictor of cardiovascular disease in the pediatric population [4,9]. Moreover, few studies have investigated the association between microalbuminuria and surrogate marker of insulin resistance such as hemoglobin A1c (HbA1c), TG/high-density lipoprotein cholesterol (HDL-C) ratio and serum alanine transaminase (ALT) in pediatric population, which have been widely accepted as risk factors of microalbuminuria in adults [10–13]. Therefore, there is, at present, no well supported evidence to support the routine measurement of microalbuminuria in the pediatric population that presents with obesity or cardiometabolic risk factors.

The aims of this study are as follows: (1) to evaluate the prevalence of microalbuminuria; (2) to assess the potential association between obesity and microalbuminuria; and (3) to examine the determinants of microalbuminuria. This study focused on the general population of Korean children and adolescents by analyzing data from the Korean National Health and Nutrition Examination Survey (KNHANES).

## Materials and methods

### Study participants

This study examined data obtained from the second and third year of the fifth KNHANES as well as the first and the second year of the sixth KNAHNES (2011–2014). The KNHANES is a nationally representative surveillance system, which has been conducted cross-sectionally since 1998 by the Korea Centers for Disease Control and Prevention and the Ministry of Health and Welfare [14]. A multi-stage clustered probability design was applied for KNHANES sampling among non-institutionalized Korean citizens. Detailed methods for KNHANES data collection are described elsewhere [15].

**Fig 1:**
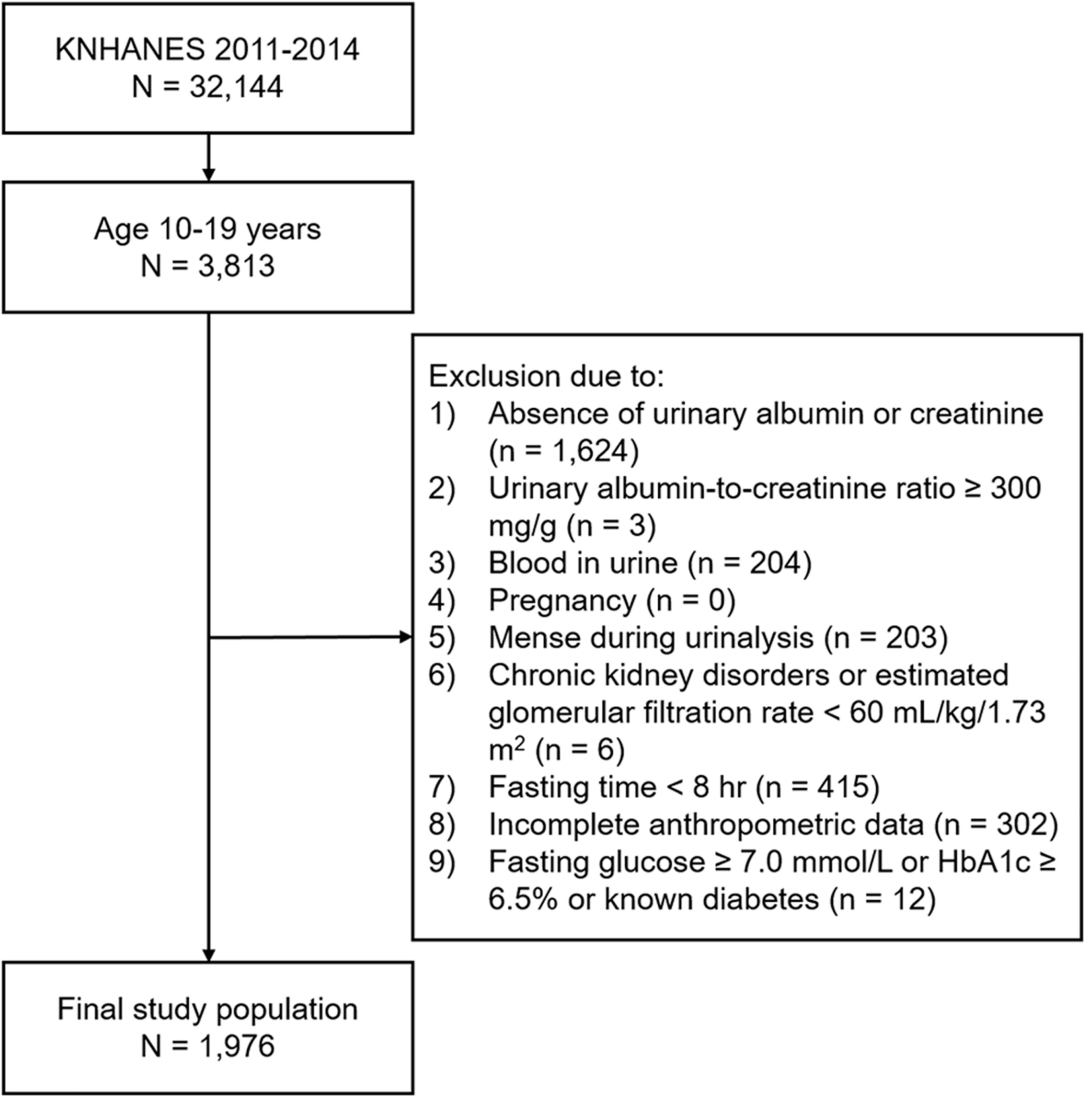
Flow chart of eligible study population.

A total of 32,144 individuals were enrolled in the KNHANES during 2011−2014. In the present study, 3,813 subjects (males 1,997 and female 1,816) aged 10–19 years were considered as potential participants. Among them, 1,837 were excluded as a result of one or more of the following: absence of urinary albumin or urinary creatinine (n = 1,624); urinary albumin to creatinine ratio ≥ 300 mg/g (n = 3); urinary blood ≥ 2+ (n = 204); pregnancy (n = 0); menstruation during urinalysis (n = 203); chronic kidney disorders or estimated glomerular filtration rate (eGFR) < 60 mL/min/1.73 m^2^ (n = 6); fasting time < 8 hours (n = 415); incomplete anthropometric data (n = 302); fasting glucose ≥ 7.0 mmol/L or HbA1c ≥ 6.5% or known diabetes (n = 12). In total, 1,976 participants (males 1,128 and females 848) met the necessary conditions and were included in the present study (Fig 1).

Informed consent was obtained from the all participants in the KNHANES. The protocol of the KNHANES was approved by the institutional review board of the Korea Centers for Disease Control and Prevention (2011-02CON-06-C, 2012-01EXP-01-2C, 2013-07-CON-03-4C, 2013-12EXP-03-5C).

### Anthropometric measurements

Anthropometric measurements were performed on all participants by trained personnel. Height was determined to the nearest 0.1 cm using a stadiometer (Seca 225, Seca, Hamburg, Germany). Weight was measured to the nearest 0.1 kg using an electronic balance (GL-6000-20, G-tech, Seoul, Korea). BMI was calculated as weight (kg) divided by height squared (m^2^), which was then transformed to a standard deviation score (SDS) using the 2007 Korean National Growth Chart [16]. The WC was measured using a flexible tape, and determined to the nearest 0.1 cm at the midpoint between the lowest margin of the rib and the uppermost border of the iliac crest during expiration (Seca 220, Seca, Hamburg, Germany). Blood pressure (BP) was measured using a mercury sphygmomanometer after the subject had rested for 5 min in a sitting position. All BP measurements were taken on the right arm three times [Baumanometer Desk model 0320 in 2011–2012 and Baumanometer Wall Unit 33(0850) in 2013–2014, W.A.Baum, New York, USA] with cuff appropriate for arm circumference. The average value of the second and third measurements of systolic BP and diastolic BP were used for subsequent analyses.

### Laboratory tests

Participant blood samples were collected by trained nurses following overnight fasting. Drawn samples were adequately prepared and transported to the Central Laboratory after proper preparation and analyzed within 24 hours. Plasma glucose, total cholesterol, HDL-C, TG, aspartate aminotransferase (AST), ALT, blood urea nitrogen (BUN) and creatinine were measured using a Hitachi Automatic Analyzer 7600 (Hitachi, Tokyo, Japan). The level of HbA1c was measured using high performance liquid chromatography (HLC-723G7; Tosoh, Tokyo, Japan), which is the method that is certified by the National Glycohemoglobin Standardization Program.

A random 20–30 mL of midstream voided urine was collected in the morning, and then used to determine its level of urinary albumin and creatinine. Urinary albumin was measured using the turbidometric method (Roche, Germany) and urinary creatinine was measured using the Jaffe rate-blanked and compensated method (Wako, Japan) with a Hitachi Automatic Analyzer 7600 (Hitachi, Tokyo, Japan). The urinary albumin to urinary creatinine ratio (UACR) was reported as milligrams of urinary albumin per grams of urinary creatinine (mg/g). The eGFR was calculated using the Bedside Schwartz equation; eGFR (mL/min/1.73 m^2^) = 0.413 x height (cm) / serum creatinine (mg/dL) [17].

### Definition of cardiometabolic risk factors and microalbuminuria

Obesity was diagnosed when a patient’s BMI was either ≥ 95^th^ percentile (depending on sex and age) or ≥ 25 kg/m^2^ [16]. Cardiometabolic risk factors were defined using the metabolic syndrome criteria set out by the international diabetes federation; systolic BP or diastolic BP ≥ 130/85 mm Hg; fasting plasma glucose ≥ 5.6 mmol/L; TG ≥ 150 mg/dL; HDL-C < 40 mg/dL in boys and girls between 10 and 15 years of age, < 50 mg/dL in girls between 16 and 19 years of age [18]. HbA1c, TG/HDL-C ratio and serum ALT were used as alternative cardiometabolic risk factors for the analysis. Elevated ALT was defined as an ALT value of > 35 IU/L for boys and > 24 IU/L for girls according to the Korean reference data [19]. Microalbuminuria was defined as a UACR of ≥ 30 mg/g and < 300 mg/g. A UACR of < 30 mg/g was considered normal.

### Statistical analyses

Statistical analyses were performed using Stata 14.2 software (StataCorp LP, College Station, Texas, USA). Appropriate sample weights were applied for the entire analyses. Results are presented as either the weighted mean ± standard error (SE) or the number of cases (weighted %). Total cholesterol, HDL-C, TG, TG/HDL-C ratio, urine microalbumin and UACR were log transformed for analyses and presented as the geometric mean ± SE. The Student’s *t*-test and chi-square test were used to compare means and proportions between groups. Logistic regression analysis was performed in order to calculate the relevant odds ratio (OR) with 95% confidence interval (CI) for a possible association between microalbuminuria and other risk factors with an adjustment for age and sex. Backward stepwise selection was used to choose the best subset of variables. Wald test was used to construct the best model. A *p*-value of < 0.05 was considered statistically significant.

## Results

### Characteristics of study participants and urinary albumin excretion

Among the 1,976 total participants included in the present study, 226 (12.2%) were obese after application of sampling weights. Fifty-eight (3.3%) presented with hypertension and 38 (2.0%) fulfilled the criteria for metabolic syndrome. In the obese group, males were disproportionately represented and the average age was higher compared to the non-obese group. In the obese group, BMI SDS, systolic BP, diastolic BP, HbA1c, total cholesterol, TG, AST, and ALT were all significantly higher compared to the non-obese group. In the non-obese group, HDL-C and eGFR were significantly higher compared to the obese group (Table 1). The proportion of subjects with metabolic syndrome was also significantly higher in the obese group (14.7% vs 0.3%, *P* < 0.001).

**Table 1:** Clinical characteristics of study participants.

	Total	Non-obese	Obese	*P*
Number (%)	1976 (100%)	1750 (87.8%)	226 (12.2%)	-
Estimated population	3,479,025	3,053,049	425,976	-
Sex (male, %)	1128 (57.1%)	975 (55.4%)	153 (68.7%)	0.001
Age (years)	14.6 ± 0.1	14.5 ± 0.1	15.7 ± 0.2	<0.001
Body mass index (kg/m^2^)	20.8 ± 0.1	19.8 ± 0.1	28.1 ± 0.2	<0.001
Body mass index SDS	0.00 ± 0.03	-0.25 ± 0.03	1.82 ± 0.04	<0.001
Waist circumference (cm)	70.2 ± 0.3	67.7 ± 0.2	88.0 ± 0.6	<0.001
Systolic blood pressure (mm Hg)	107.8 ± 0.3	106.7 ± 0.3	116.0 ± 0.8	<0.001
Diastolic blood pressure (mm Hg)	66.2 ± 0.3	65.8 ± 0.3	69.6 ± 0.7	<0.001
Fasting glucose (mmol/L)	5.0 ± 0.1	5.0 ± 0.1	5.0 ± 0.3	0.178
Hemoglobin A1c (%)	5.46 ± 0.01	5.45 ± 0.01	5.52 ± 0.02	0.002
Total cholesterol (mg/dL)	156.3 ± 0.8	155.7 ± 0.8	160.8 ± 2.3	0.034
HDL-cholesterol (mg/dL)	49.9 ± 0.3	50.9 ± 0.3	43.7 ± 0.6	<0.001
TG (mg/dL)	73.3 ± 1.1	70.3 ± 1.1	98.2 ± 3.8	<0.001
TG/HDL-C ratio	1.47 ± 0.02	1.38 ± 0.02	2.25 ± 0.10	<0.001
Aspartate transaminase (IU/L)	18.9 ± 0.2	18.4 ± 0.2	22.4 ± 0.9	<0.001
Alanine transaminase (IU/L)	15.6 ± 0.4	13.5 ± 0.3	30.5 ± 2.4	<0.001
Urine microalbumin (μg/mL)	5.4 ± 0.2	5.7 ± 0.3	4.0 ± 0.5	0.004
Urine creatinine (mg/dL)	206.0 ± 2.8	204.4 ± 3.0	218.0 ± 8.1	0.109
UACR (mg/g creatinine)	3.0 ± 0.1	3.2 ± 0.1	2.1 ± 0.2	<0.001
Microalbuminuria (%)	59 (3.0%)	56 (3.2%)	3 (1.7%)	0.238
eGFR (mL/min/1.73 m^2^)	94.2 ± 0.5	94.7 ± 0.5	90.3 ± 1.2	<0.001
Abdominal obesity (%)	172 (9.3%)	37 (2.2%)	135 (60.2%)	<0.001
Hypertension (%)	58 (3.3%)	33 (1.9%)	25 (13.6%)	<0.001
Hyperglycemia (%)	326 (16.8%)	295 (17.1%)	31 (14.8%)	0.424
Hypertriglyceridem (%)ia	324 (16.4%)	262 (14.8%)	62 (27.7%)	<0.001
Low HDL-cholesterol (%)	275 (14.7%)	199 (12.0%)	76 (34.1%)	<0.001
Metabolic syndrome (%)	38 (2.0%)	5 (0.3%)	33 (14.7%)	<0.001

Data were expressed as weighted mean ± standard error or number of cases (weighted %).

SDS, standard deviation score; HDL-C, high-density lipoprotein cholesterol; TG, triglyceride; UACR, urinary albumin-to-creatinine ratio; eGFR, estimated glomerular filtration rate.

Total cholesterol, HDL-cholesterol, triglyceride, triglyceride/HDL-cholesterol ratio, urine microalbumin and urinary albumin-to-creatinine ratio were log transformed for analysis and expressed as geometric mean ± standard error.

**Fig 2:**
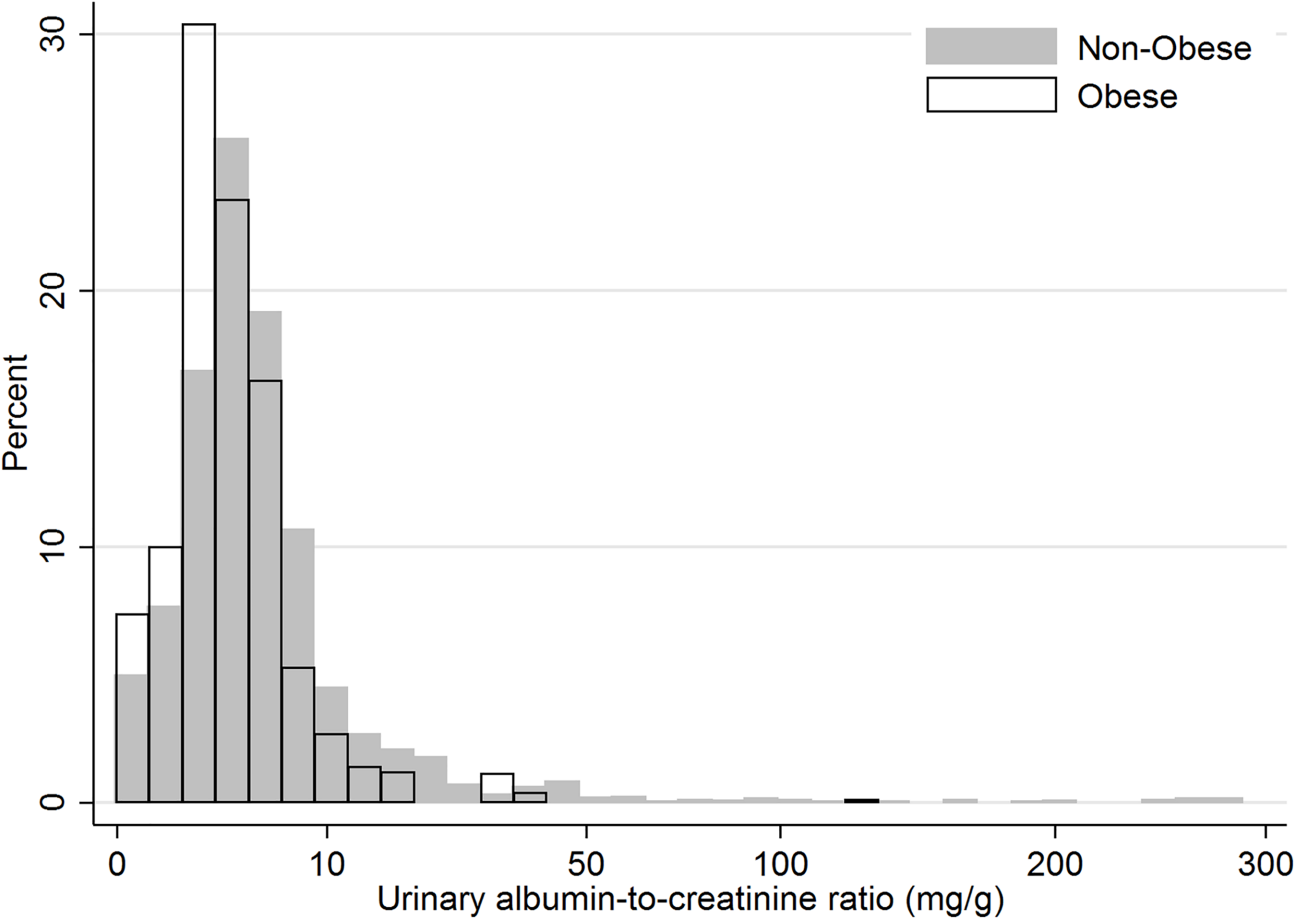
Distribution of urinary albumin-to-creatinine ratio by body mass index category.

**Fig 3:**
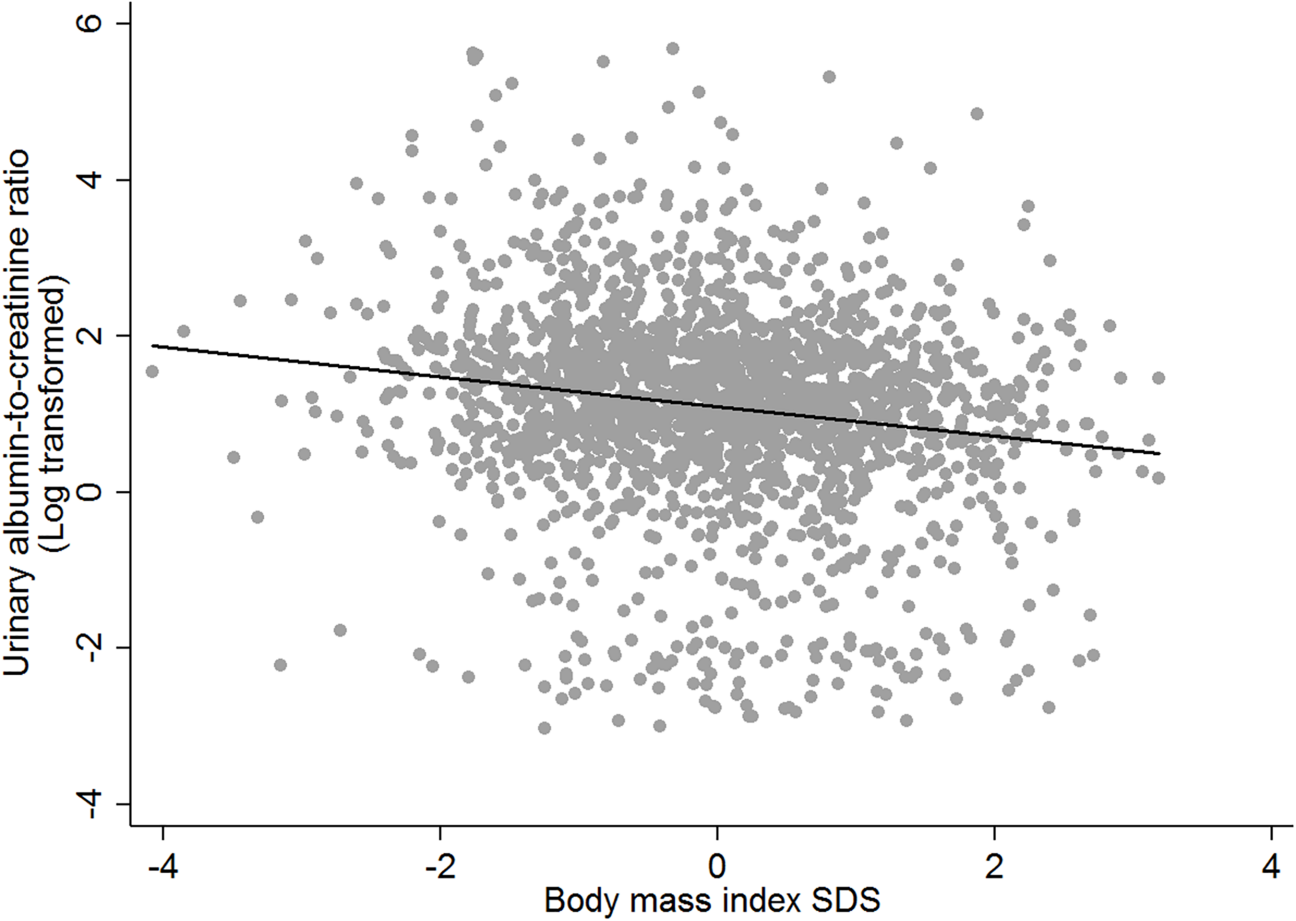
The relationship between the log-transformed urinary albumin-to-creatinine ratio (mg/g creatinine) and body mass index SDS (coefficient = -0.19, *P* < 0.001).

The prevalence of microalbuminuria was 3.0%, and the distribution of UACR across all subjects by BMI category included in this study is presented in Fig 2. In the non-obese group, the microalbuminuria prevalence was greater compared to the obese group, though this difference was not statistically significant (3.2% vs. 1.7%; *P* = 0.238). The mean UACR for non-obese youth was significantly greater compared to that for obese youth (3.2 ± 0.1 vs. 2.1 ± 0.2 mg/g; *P* < 0.001). In the regression analysis, negative correlation was observed between BMI SDS and logarithmic UACR (coefficient -0.19, *P* < 0.001, Fig 3.).

### Comparison between the microalbuminuria and normoalbuminuria groups

We found that BMI SDS was significantly lower in the microalbuminuria group compared to the normoalbuminuria group with application of sampling weights (Table 2). HbA1c (%) was significantly higher in the microalbuminuria group (5.46 ± 0.01 for the normoalbuminuria group and 5.56 ± 0.04 for microalbuminuria group; *P* = 0.015). HDL-C were significantly lower in the normoalbuminuria group (49.8 ± 0.3 mg/dL for the normoalbuminuria group and 54.0±1.6 mg/dL for microalbuminuria group; *P* = 0.007). There was no difference, however, in levels of metabolic syndrome and its components between the two groups, with the exception of HDL-C (*P* = 0.006).

**Table 2:** Clinical characteristics of subjects according to microalbuminuria.

	Normoalbuminuria	Microalbuminuria	*P*
N (%)	1,917 (97.0%)	59 (3.0%)	-
Estimated population	3,374,504	104,521	-
Sex (male, %)	1,095 (56.9%)	33 (61.5%)	0.547
Age (yr)	14.6 ± 0.1	14.1 ± 0.4	0.248
Body mass index (kg/m^2^)	20.9 ± 0.1	19.3 ± 0.7	0.029
Body mass index SDS	0.02 ± 0.03	-0.51 ± 0.21	0.011
Waist circumference (cm)	70.3 ± 0.3	67.1 ± 1.8	0.077
Systolic blood pressure (mm Hg)	107.8 ± 0.3	108.4 ± 1.8	0.729
Diastolic blood pressure (mm Hg)	66.2 ± 0.3	68.2 ± 1.5	0.164
Fasting glucose (mmol/L)	5.0 ± 0.0	5.0 ± 0.1	0.795
Hemoglobin A1c (%)	5.46 ± 0.01	5.56 ± 0.04	0.015
Total cholesterol (mg/dL)	156.2 ± 0.8	159.6 ± 6.2	0.582
HDL-C (mg/dL)	49.8 ± 0.3	54.0 ± 1.6	0.007
TG (mg/dL)	73.4 ± 1.1	72.4 ± 6.0	0.873
TG/HDL-C ratio	1.47 ± 0.02	1.34 ± 0.14	0.357
Aspartate transaminase (IU/L)	18.9 ± 0.2	19.8 ± 1.6	0.577
Alanine transaminase (IU/L)	15.4 ± 0.4	20.5 ± 6.7	0.453
Serum BUN (mg/dL)	11.9 ± 0.1	12.7 ± 0.8	0.322
Serum creatinine (mg/dL)	0.74 ± 0.00	0.76 ± 0.03	0.568
Urine microalbumin (μg/mL)	5.0 ± 0.2	103.3 ± 18.4	<0.001
Urine creatinine (mg/dL)	206.1 ± 2.8	205.1 ± 21.0	0.964
UACR (mg/g creatinine)	2.7 ± 0.1	65.4 ± 6.4	<0.001
eGFR (mL/min/1.73 m^2^)	94.2 ± 0.5	93.2 ± 2.8	0.717
Abdominal obesity (%)	169 (9.4%)	3 (6.8%)	0.572
Hypertension (%)	57 (3.3%)	1 (4.6%)	0.774
Hyperglycemia (%)	314 (16.6%)	12 (23.4%)	0.348
Hypertriglyceridemia (%)	317 (16.5%)	7 (13.5%)	0.620
Low HDL-cholesterol (%)	272 (15.0%)	3 (5.2%)	0.006
Metabolic syndrome (%)	37 (1.9%)	1 (4.6%)	0.557

Data were expressed as weighted mean ± standard error or number of cases (weighted %).

SDS, standard deviation score; HDL-C, high-density lipoprotein cholesterol; TG, triglyceride; BUN, blood urea nitrogen; UACR, urinary albumin-to-creatinine ratio; eGFR, estimated glomerular filtration rate.

Total cholesterol, HDL-cholesterol, triglyceride, triglyceride/HDL-cholesterol ratio, urine microalbumin and urinary albumin-to-creatinine ratio were log transformed for analysis and expressed as geometric mean ± standard error.

### Factors associated with microalbuminuria

We observe that HbA1c was associated with microalbuminuria in both groups with an adjustment for age and sex [adjusted OR 3.77 (95% CI 1.08–13.12) in the non-obese group and 5.27 (95% CI 2.11–13.22) in the obese group] after applying sampling weights. In the obese group, the presence of hypertension and metabolic syndrome was a significant predictor of microalbuminuria [adjusted OR 19.53 (95% CI 1.11–341.92) and 16.00 (95% CI 1.07–239.53), respectively] (Table 3). In the obese group, the high level of TG/HDL-C ratio were also a significant predictor of microalbuminuria, of which adjusted OR was 1.31 (95% CI 1.04–1.64; *P* = 0.022). However, high ALT, hypertriglyceridemia and low HDL-C were not associated with microalbuminuria in both groups. After backward stepwise selection of variables best models were constructed. In the non-obese group, OR of hyperglycemia and HbA1c were 2.62 (95% CI 1.09–6.30) and 3.34 (95% CI 1.09–10.17), respectively. In the obese group, OR of the presence of hypertension and HbA1c was 14.10 (95% CI 1.12–177.98) and 6.68 (95% CI 1.87–23.95), respectively.

**Table 3:** Odds ratio for the occurrence of microalbuminuria and cardiometabolic risk factors according to BMI category.

Group	Variables	Unadjusted OR (95% CI)	*P*	Adjusted OR (95% CI)^a^	*P*	OR (95% CI)^b^	*P*
Non-obese	Abdominal obesity^c^	-	-	-	-	-	-
	Hypertension^c^	-	-	-	-	-	-
	Hyperglycemia	1.65 (0.76, 3.62)	0.208	1.54 (0.73, 3.26)	0.260	2.62 (1.09, 6.30)	0.031
	Hypertriglyceridemia	0.60 (0.21, 1.71)	0.339	0.56 (0.20, 1.57)	0.271	-	-
	Low HDL-C	0.29 (0.07, 1.23)	0.092	0.31 (0.07, 1.33)	0.115	-	-
	Metabolic syndrome^c^	-	-	-	-	-	-
	High ALT	0.88 (0.27, 2.90)	0.836	0.82 (0.26, 2.56)	0.731	-	-
	TG/HDL-C ratio	0.79 (0.55, 1.12)	0.187	0.79 (0.56, 1.12)	0.189	-	-
	Hemoglobin A1c (%)	4.11 (1.24, 13.64)	0.021	3.77 (1.08, 13.12)	0.037	3.34 (1.09, 10.17)	0.034
Obese	Abdominal obesity^c^	-	-	-	-	-	-
	Hypertension	14.67 (1.17, 183.85)	0.037	19.53 (1.11, 341.92)	0.042	14.10 (1.12, 177.98)	0.041
	Hyperglycemia^c^	-	-	**-**	**-**	**-**	**-**
	Hypertriglyceridemia	5.74 (0.47, 70.42)	0.171	6.24 (0.73, 52.98)	0.093	-	-
	Low HDL-C	0.59 (0.04, 8.33)	0.697	0.56 (0.04, 7.37)	0.655	-	-
	Metabolic syndrome	13.37 (1.07, 166.64)	0.044	16.00 (1.07, 239.53)	0.045	-	-
	High ALT	5.09 (0.41, 62.80)	0.204	5.37 (0.61, 47.54)	0.130	-	-
	TG/HDL-C ratio	1.29 (0.95, 1.74)	0.099	1.31 (1.04, 1.64)	0.023	-	-
	Hemoglobin A1c (%)	5.16 (1.81, 14.77)	0.002	5.27 (2.11, 13.22)	<0.001	6.68 (1.87, 23.95)	0.004

^*a*^ Adjusted for age and sex

^*b*^ Odds ratios after backward stepwise selection of variables.

^*c*^ Odds ratios could not be estimated due to small sample size for one comparison group in these variables.

HDL-C, high-density lipoprotein cholesterol; ALT, alanine transaminase; TG; triglyceride

## Discussion

Using nationally representative data, the present study measured a relatively low prevalence of microalbuminuria in obese Korean children and adolescents compared to non-obese counterparts. According to the available literature, however, the prevalence of microalbuminuria in obese children ranges from 0.3% to 23.9%, with significant variation [9,20,21]. Similar to the results of this study, a recent report determined that the prevalence of microalbuminuria in obese Spanish youth was 2.4% and not prominent in obese children [6]. Goknar et al. also reported that microalbuminuria was not found to be different between obese children and healthy controls [8]. One possible explanation for this finding is that underweight children with a low muscle mass might present with the reduced excretion of urinary creatinine, thereby resulting in an increased level of UACR [22]. A previous study that examined Korean adults aged 30 years and older and without diabetes, hypertension, renal failure, or overt proteinuria, also showed that the greater prevalence of microalbuminuria is associated with underweight men [23].

In this study, we found that microalbuminuria is associated with the presence of metabolic syndrome in the obese group. Prior work has suggested that microalbuminuria in obese youth is related with cardiometabolic risk factors such as WC and the level of TG, and it is suggested that the central body fat distribution is related to renal function impairment [6]. Also consistent with our findings, we note that there is a data that suggests microalbuminuria is associated with metabolic syndrome in obese children and adolescents [24]. Additionally, there are reports that obese adolescents with metabolic syndrome exhibit a significantly reduced glomerular filtration rate compared with obese adolescents that do not present with metabolic syndrome. Together, this suggests that metabolic syndrome can increase the risk of kidney dysfunction in obese adolescents [25]. The definition of metabolic syndrome in pediatric population varies across different published studies, and there have been many opinions that microalbuminuria is a component of metabolic syndrome. Our data support these opinions, and additional strategies going forward that will prevent the renal complications in children with metabolic syndrome should be implemented. Abdominal obesity is a crucial component of metabolic syndrome, which has been reported to be more strongly associated with microalbuminuria rather overall obesity in general, and in our study, although BMI did not show a significant association, metabolic syndrome was positively and significantly associated with microalbuminuria in the pediatric population with obesity [26]. Abdominal obesity, measured using WC, has been known to be a reliable surrogate marker for visceral fat deposition in both children and adults, which was superior to BMI in predicting cardiometabolic risk factors [27]. It has been suggested that adiponectin, an anti-inflammatory substance, is decreased in subjects presenting with high levels of visceral fat, which can in turn lead to inflammation-induced microalbuminuria and cardiovascular disease [28]. In the present study, association between microalbuminuria and WC could not be evaluated due to the small number of subjects with microalbuminuria. However, WC should be measured in the general pediatric population, and particularly obese children and adolescents because metabolic syndrome, which includes WC as one of diagnostic criteria, was an important clinical predictor of microalbuminuria.

In this study, microalbuminuria was associated with the level of HbA1c in both the obese and non-obese groups. To the best of our knowledge, this is the first research which showed association between HbA1c and microalbuminuria in children and adolescents without diabetes mellitus. Elevated HbA1c is associated with many cardiovascular risk factors and surrogate markers of insulin resistance. Chen and colleagues have reported that high levels of HbA1c are associated with the subclinical atherosclerosis in obese Chinese children without diabetes [29]. In adult population HbA1c are associated with diabetes, cardiovascular disease and death [30]. Another recent support argues that HbA1c is associated with low-grade albuminuria in Chinese adults, and it has been suggested that insulin resistance could result in endothelial dysfunction and the dysfunction of the glomerular capillary wall leading to albuminuria [10]. Bartz et al. also demonstrated that the adiposity-related insulin resistance to endothelial function may impact renal dysfunction, and so UACR provides an early marker of endothelial dysfunction in obese adolescents [31]. Additionally, this study sought to perform the assessment of endothelial function by peripheral arterial tonometry, and suggests that endothelial dysfunction mediates the link between obesity-related insulin resistance and early microalbuminuria [31]. The present study also suggests that insulin resistance may play an important role in the development of renal impairment in conjunction with central obesity, which was a crucial component of diagnosis of metabolic syndrome among children and adolescents. In the present study, TG/HDL-C ratio was associated with presence of microalbuminuria in obese youths. TG/HDL-C ratio was a recently introduced non-conventional lipid profile, which reflects small, dense low-density-lipoprotein particles [32]. It was known to be a good predictor of cardiovascular disease and mortality in adults [33,34]. It was associated with insulin resistance in obese youths, which was suggested as the method to identify subjects at risk [35]. Therefore, elevated TG/HDL-C ratio may reflect endothelial dysfunction caused by insulin resistance, which resulted in microalbuminuria.

In this study, we found that microalbuminuria was associated with hypertension in the obese group. Microalbuminuria has been found in 8–15% of patients with primary hypertension, and it had previously been suggested that blood pressure load may cause endothelial dysfunction resulting in increased systemic vascular permeability and the development of microalbuminuria [4]. Prior studies have suggested that microalbuminuria might provide an integrated marker for cardiovascular risk and target organ damage in hypertensive patients [4,9,36]. The Heart Outcomes Prevention Evaluation found that major cardiovascular events increased by 5.95 for every 3.0 mg/g increase in UACR [37]. Nguyen et al. also reported that there was a significant association between hypertension and microalbuminuria in obese adolescents [9]. The results of the present study largely support the conclusions of the previous studies cited here, and BP might be controlled in order to prevent renal and cardiovascular diseases from developing in other obese children.

This study had several limitations. Firstly, it would be possible that orthostatic proteinuria was included in our subjects. However, only a small portion of the study participants may be affected because urinary samples were collected in the morning. Secondly, UACR was not measured repeatedly in subjects with microalbuminuria, which might cause misclassification bias. Thirdly, normal-weight children and adolescents are more likely to be active and exercise prior to urine collection, and as such the active exercise might lead to physiological microalbuminuria. Fourthly, data were collected cross-sectionally, which means that it might not reflect long-term effect of high BMI to development of microalbuminuria. Fifthly, body composition was not considered for analyses because of lack of data. BMI itself could not differentiate between fat and lean mass. Microalbuminuria may be affected by lean mass, which accounts for urine creatinine excretion.

In conclusion, we identify a low prevalence of microalbuminuria in obese Korean children and adolescents. Our findings demonstrated that the presence of hypertension and hyperglycemia was associated with microalbuminuria and suggested that additional monitoring and intervention for microalbuminuria should be undertaken, to prevent future cardiovascular events. Especially Hemoglobin A1c was associated with microalbuminuria in youths regardless of weight status. In obese children, hypertension has been associated with renal injury, and we suggest that blood pressure should be carefully monitored and controlled. Therefore, our data support the measurement of microalbuminuria in children with abnormal glucose metabolism and obese children with hypertension for the assessment of cardiovascular risk.
